# Supplementary material for: Actin waves guide an outward movement of microclusters in the lymphocyte immunological synapse
Source: EMBO Rep. 2025 Dec 22;27(4):834–52. doi: 10.1038/s44319-025-00676-2 (PMC12936205; doi:10.1038/s44319-025-00676-2)
Supplement: Supplementary file 1 — Table EV1 [file 44319_2025_676_MOESM1_ESM.docx]

**Table EV1:** p-values associated with Figure 3B – A - represents p-values associated with TCR anterograde fraction; B - represents p-values associated with actin anterograde fraction; C - represents p-values associated with TCR speed; D – represents p-values associated with actin flow speed.

|  | |  |  |
| --- | --- | --- | --- |
| A | p values TCR anterograde fraction (top left graph): |  |  |
|  | Primary WASP-/- vs. Primary Control |  | 2.49E-03 |
|  | Primary WASP-/- vs. Primary CK666 |  | 9.31E-04 |
|  | Primary Control vs. Primary CK666 |  | 9.64E-01 |
|  |  |  |  |
|  |  |  |  |
| B | p values actin anterograde fraction (Bottom left graph): |  |  |
|  | Primary CK666 vs. Primary WASP-/- |  | 0.132318 |
|  | Primary CK666 vs. Primary Control |  | 0.094369 |
|  | Primary WASP-/- vs. Primary Control |  | 0.302475 |
|  |  |  |  |
|  |  |  |  |
| C | p values TCR speed (Top right graph): |  |  |
|  | Mouse WASP-/- vs. Primary Control |  | 0.306625 |
|  | Mouse WASP-/- vs. Primary CK666 |  | 0.878477 |
|  | Mouse CK666 vs. Primary Control |  | 0.287314 |
|  |  |  |  |
|  |  |  |  |
| D | p values actin speed (bottom right graph): |  |  |
|  | Primary CK666 vs. Primary WASP-/- |  | 0.001437 |
|  | Primary CK666 vs. Primary Control |  | 0.33657 |
|  | Primary WASP-/- vs. Primary Control |  | 0.003976 |
