# Supplementary material for: Actin waves guide an outward movement of microclusters in the lymphocyte immunological synapse
Source: EMBO Rep. 2025 Dec 22;27(4):834–52. doi: 10.1038/s44319-025-00676-2 (PMC12936205; doi:10.1038/s44319-025-00676-2)
Supplement: Supplementary file 3 — Movie EV1 [file 44319_2025_676_MOESM3_ESM.zip › Movie EV1/Movie EV1.docx]

**Movie EV1.** Comparison of manual (left panel) and automated (Right panel) tracking of TCR clusters in Jurkat T cells. This movie corresponds to Figure EV1. The manual vs automated speeds were found to be comparable (manual: 45.72 ± 7 nm/sec; automated: 48.26 ± 9 nm/sec).
