# Supplementary material for: Actin waves guide an outward movement of microclusters in the lymphocyte immunological synapse
Source: EMBO Rep. 2025 Dec 22;27(4):834–52. doi: 10.1038/s44319-025-00676-2 (PMC12936205; doi:10.1038/s44319-025-00676-2)
Supplement: Supplementary file 4 — Movie EV2 [file 44319_2025_676_MOESM4_ESM.zip › Movie EV2/Movie EV2.docx]

**Movie EV2.** Automated tracking of TCR clusters in Jurkat T cells. The left panel shows raw images, while the panel on the right shows positional color-coded trajectories. The movie corresponds to Figure 1C.
