# Supplementary material for: Actin waves guide an outward movement of microclusters in the lymphocyte immunological synapse
Source: EMBO Rep. 2025 Dec 22;27(4):834–52. doi: 10.1038/s44319-025-00676-2 (PMC12936205; doi:10.1038/s44319-025-00676-2)
Supplement: Supplementary file 5 — Movie EV3 [file 44319_2025_676_MOESM5_ESM.zip › Movie EV3/Movie EV3.docx]

**Movie EV3.** Automated tracking of TCR clusters in mouse Primary T cells. The left panel shows raw images, while the panel on the right shows positional color-coded trajectories. The movie corresponds to Figure 1C.
