# Supplementary material for: Actin waves guide an outward movement of microclusters in the lymphocyte immunological synapse
Source: EMBO Rep. 2025 Dec 22;27(4):834–52. doi: 10.1038/s44319-025-00676-2 (PMC12936205; doi:10.1038/s44319-025-00676-2)
Supplement: Supplementary file 6 — Movie EV4 [file 44319_2025_676_MOESM6_ESM.zip › Movie EV4/Movie EV4.docx]

**Movie EV4.** Inset showing magnified details of TCR trajectories from Movie 3. The movie corresponds to Figure 1C.
