# Supplementary material for: Actin waves guide an outward movement of microclusters in the lymphocyte immunological synapse
Source: EMBO Rep. 2025 Dec 22;27(4):834–52. doi: 10.1038/s44319-025-00676-2 (PMC12936205; doi:10.1038/s44319-025-00676-2)
Supplement: Supplementary file 8 — Movie EV6 [file 44319_2025_676_MOESM8_ESM.zip › Movie EV6/New Microsoft Word Document.docx]

**Movie EV6.** PIV flow analysis of LifeAct-GFP in mouse Primary T cells. The left panel shows LifeAct-GFP distribution, while the panel of the right panel shows corresponding PIV flow vectors (pseudocolored green) overlaid on LifeAct-GFP (greyscale) at the given time points. This video corresponds to Figure 2A.
