# Supplementary material for: Actin waves guide an outward movement of microclusters in the lymphocyte immunological synapse
Source: EMBO Rep. 2025 Dec 22;27(4):834–52. doi: 10.1038/s44319-025-00676-2 (PMC12936205; doi:10.1038/s44319-025-00676-2)
Supplement: Supplementary file 9 — Movie EV7 [file 44319_2025_676_MOESM9_ESM.zip › Movie EV7/Movie EV7.docx]

**Movie EV7.** Actin wavefront detection and corresponding TCR microcluster trajectories in a Primary T cell. The left panel shows TCR (pseudocolored red) and LifeAct (pseudocolored green) distribution in raw images, while the panel on the right shows an overlay of detected actin wavefronts (green lines) and TCR trajectories (positional color-coded tracks). This video corresponds to Figure 2C.
