# Supplementary material for: Actin waves guide an outward movement of microclusters in the lymphocyte immunological synapse
Source: EMBO Rep. 2025 Dec 22;27(4):834–52. doi: 10.1038/s44319-025-00676-2 (PMC12936205; doi:10.1038/s44319-025-00676-2)
Supplement: Supplementary file 10 — Movie EV8 [file 44319_2025_676_MOESM10_ESM.zip › Movie EV8/Movie EV8.docx]

**Movie EV8.** Wavefront movement (green lines), corresponding PIV actin flow vectors (green arrows), and TCR trajectories (red lines) in a Primary T cell.
