# Supplementary material for: Actin waves guide an outward movement of microclusters in the lymphocyte immunological synapse
Source: EMBO Rep. 2025 Dec 22;27(4):834–52. doi: 10.1038/s44319-025-00676-2 (PMC12936205; doi:10.1038/s44319-025-00676-2)
Supplement: Supplementary file 11 — Movie EV9 [file 44319_2025_676_MOESM11_ESM.zip › Movie EV9/Movie EV9.docx]

**Movie EV9.** A movie of the mechanistic model 1 (“mode 1”) to explore actin wavefronts (green lines) – TCR tracer (red /blue balls) association. The model shows dynamics of actin wavefronts from experimental data, overlaid with particles representing anterogradely moving tracers (red) and the tracer particles undergoing a default retrograde trajectory to the cSMAC (blue). For details see methods section. This video corresponds to Figure 2E top panel.
