# Supplementary material for: Actin waves guide an outward movement of microclusters in the lymphocyte immunological synapse
Source: EMBO Rep. 2025 Dec 22;27(4):834–52. doi: 10.1038/s44319-025-00676-2 (PMC12936205; doi:10.1038/s44319-025-00676-2)
Supplement: Supplementary file 12 — Movie EV10 [file 44319_2025_676_MOESM12_ESM.zip › Movie EV10/Movie EV10.docx]

**Movie EV10.** A movie of the mechanistic model 2 (“mode 2”) that overlays TCR tracers (red/blue balls) on PIV actin flows (green arrows). This video corresponds to Figure 2E bottom panel. Red- Outward moving tracers; Blue- Inward moving tracers.
