# Supplementary material for: Actin waves guide an outward movement of microclusters in the lymphocyte immunological synapse
Source: EMBO Rep. 2025 Dec 22;27(4):834–52. doi: 10.1038/s44319-025-00676-2 (PMC12936205; doi:10.1038/s44319-025-00676-2)
Supplement: Supplementary file 15 — Movie EV13 [file 44319_2025_676_MOESM15_ESM.zip › Movie EV13/Movie EV13.docx]

**Movie EV13.** LifeAct-GFP (top left panel, pseudocolored green) and TCR (top right panel, pseudocolored red) movement in a 50μm CK666-treated Primary T cell. The bottom left panel shows a combined distribution of the top two panels, while the bottom right panel shows positionally color-coded TCR trajectories obtained using automated tracking. The movie corresponds to Figure 3A.
