# Supplementary material for: Actin waves guide an outward movement of microclusters in the lymphocyte immunological synapse
Source: EMBO Rep. 2025 Dec 22;27(4):834–52. doi: 10.1038/s44319-025-00676-2 (PMC12936205; doi:10.1038/s44319-025-00676-2)
Supplement: Supplementary file 20 — Movie EV18 [file 44319_2025_676_MOESM20_ESM.zip › Movie EV18/Movie EV18.docx]

**Movie EV18.** PIV flow analysis of LifeAct-EGFP in mouse Primary CD8+ T cell treated with 200nM Latrunculin, from images acquired using conventional TIRF microscopy. The left panel shows LifeAct-EGFP distribution, while the panel of the right panel shows corresponding PIV flow vectors (pseudocolored green) overlaid on LifeAct-EGFP (greyscale) at the given time points. This video corresponds to Figure EV3A.
