# Supplementary material for: Actin waves guide an outward movement of microclusters in the lymphocyte immunological synapse
Source: EMBO Rep. 2025 Dec 22;27(4):834–52. doi: 10.1038/s44319-025-00676-2 (PMC12936205; doi:10.1038/s44319-025-00676-2)
Supplement: Supplementary file 22 — Movie EV20 [file 44319_2025_676_MOESM22_ESM.zip › Movie EV20/Movie EV20.docx]

**Movie EV20.** Another example of actin wavefront movement (marked by the green line in the right panel) and TCR trajectories (positionally coded trajectories derived using automated tracking; right panel) in a WASP-/- Primary T cell. The panel on the left shows the combined distribution of LifeAct (pseudocolored green) and TCR (pseudocolored red). This video corresponds to Figure 3D.
