# Supplementary material for: Actin waves guide an outward movement of microclusters in the lymphocyte immunological synapse
Source: EMBO Rep. 2025 Dec 22;27(4):834–52. doi: 10.1038/s44319-025-00676-2 (PMC12936205; doi:10.1038/s44319-025-00676-2)
Supplement: Supplementary file 27 — Expanded View Figures [file 44319_2025_676_MOESM27_ESM.pdf]

Expanded View Figures

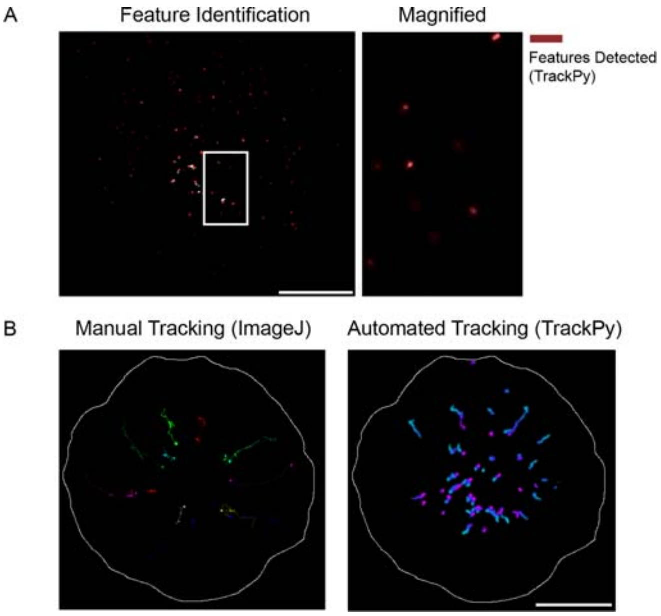

Figure EV1. Validation of automated microcluster detection and tracking methodology.

(**A**) Validation of automated TCR microcluster ("feature") detection. The position of the detected clusters are denoted in red pixels, the colocalization of the red pixels with the highest intensity pixels of the raw image (>150) was then measured and found to be 97.2%. (**B**) Validation of tracking routine. Manual tracking of TCR was performed in ImageJ where a particle trajectory was plotted by manually identifying the position of the particle at each time and then combining the identified points to form a track, the cell boundaries demarcated by white borders. The corresponding speeds of TCRs were compared and found to show similar results (manual: 45.72 ± 6.82 nm/s; automated: 48.26 ± 8.91 nm/s). Scale Bar = 5 µm. Source data are available online for this figure.

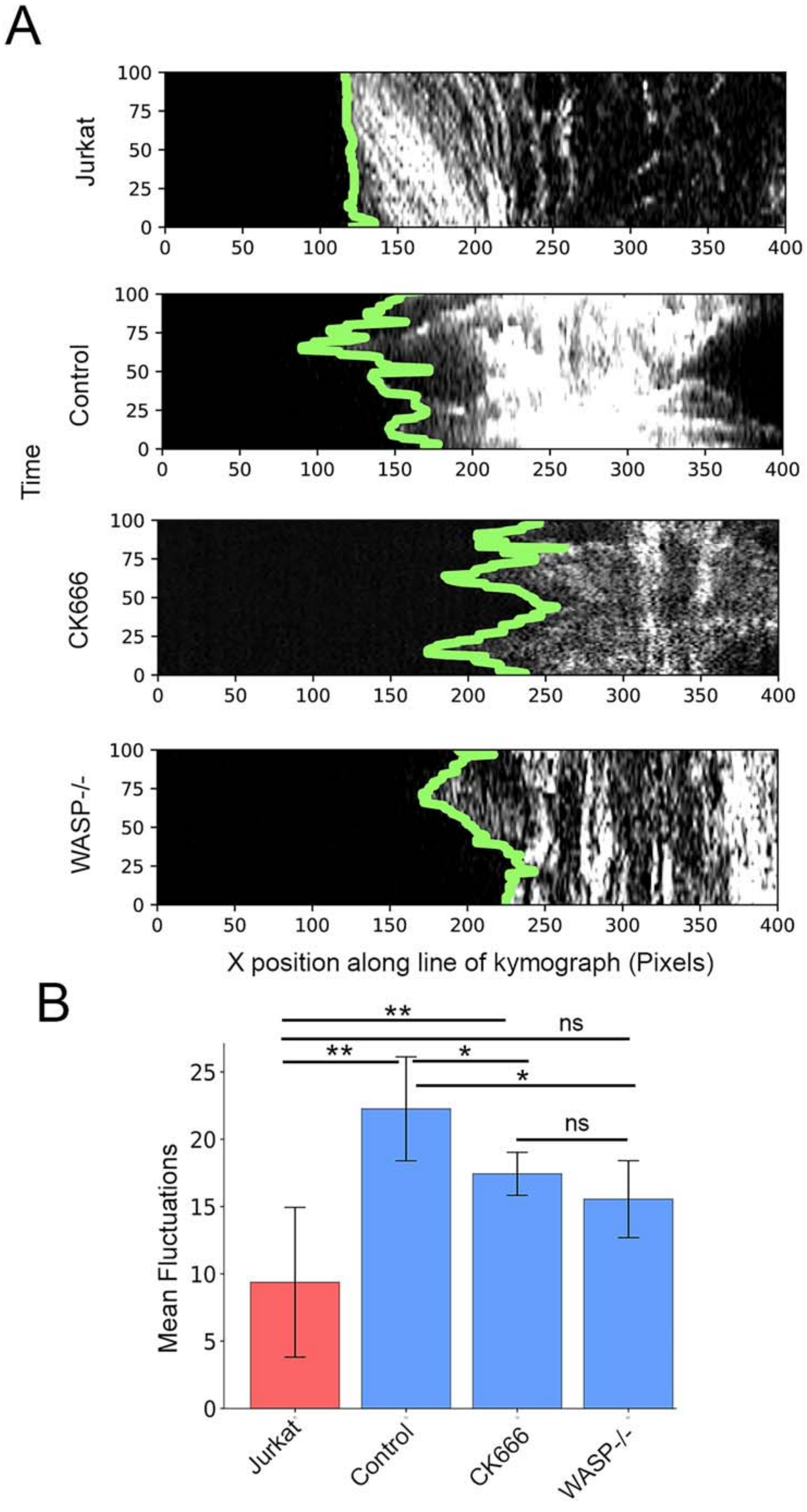
A
Jurkat
Control
CK666
WASP-/-
Time
X position along line of kymograph (Pixels)
B
Mean Fluctuations
Jurkat
Control
CK666
WASP-/-
**
ns
*

Figure EV2. Comparison of cortical lamellar waves and fluctuations at synapse.

(**A**) Kymographs showing actin waves in Jurkat (top panel) and Primary T cells (bottom three panels), the green lines indicate the evolution of actin boundary over time. (**B**) A comparison of the fluctuation from the mean of the cell edges (No. of cells analyzed $n \geq 5$) (RMS fluctuations *p*-values: Jurkat vs. Primary control- 0.001; Jurkat vs. Primary CK666- 0.006; Jurkat vs. Primary WASP-/- 0.072; Primary control vs. Primary CK666- 0.018; Primary control vs. Primary WASP-/- 0.017; Primary CK666 vs. Primary WASP-/- 0.214) (Data represented as Mean ± SEM). Source data are available online for this figure.

© The Author(s)

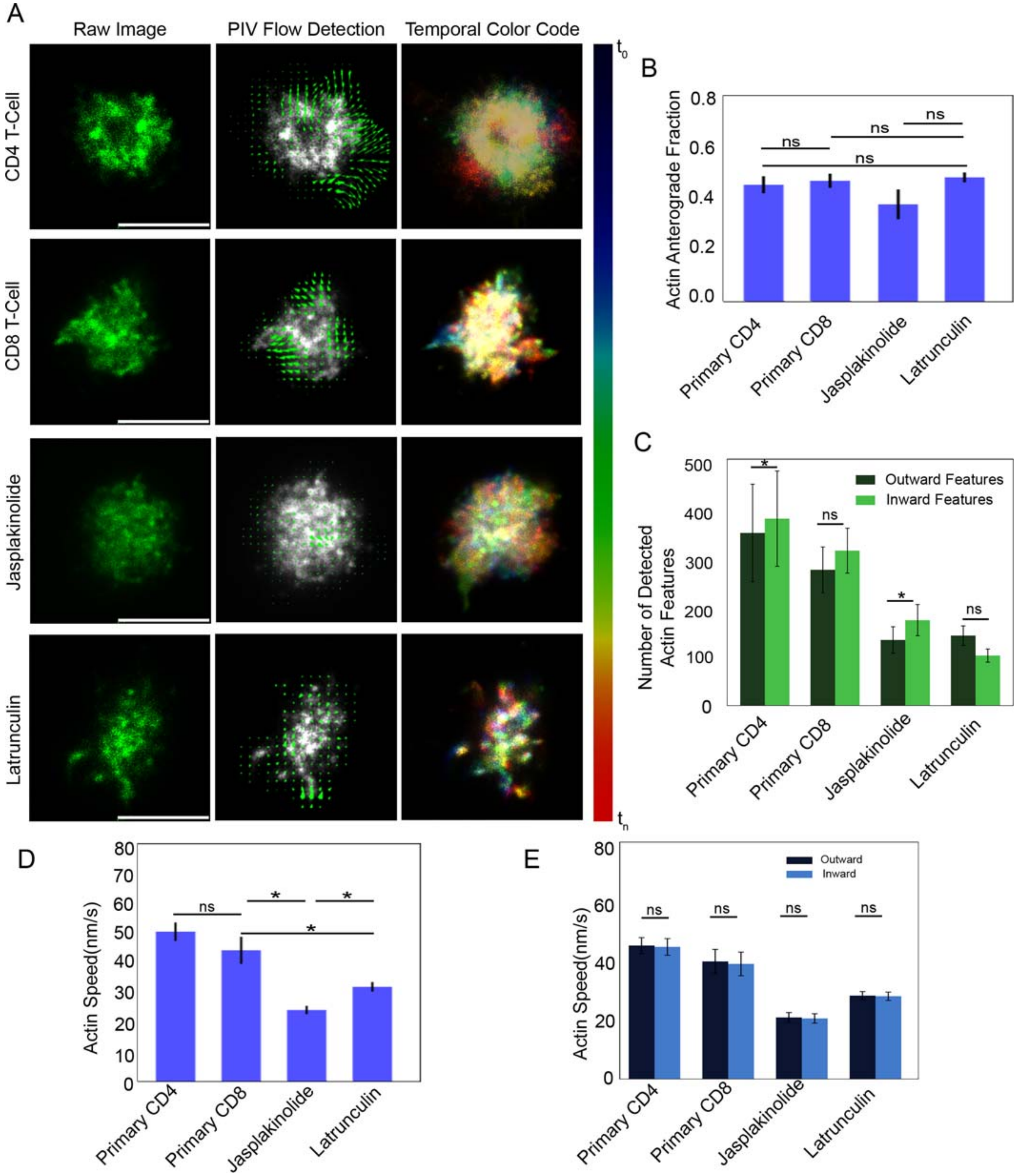
A
Raw Image
PIV Flow Detection
Temporal Color Code
CD4 T-Cell
CD8 T-Cell
Jasplakinolide
Latrunculin
t_0
t_n
B
Actin Anterograde Fraction
0.8
0.6
0.4
0.2
0.0
ns
ns
ns
ns
Primary CD4
Primary CD8
Jasplakinolide
Latrunculin
C
Number of Detected Actin Features
500
400
300
200
100
0
Outward Features
Inward Features
*
ns
*
ns
Primary CD4
Primary CD8
Jasplakinolide
Latrunculin
D
Actin Speed(nm/s)
80
70
60
50
40
30
20
10
0
ns
*
*
*
Primary CD4
Primary CD8
Jasplakinolide
Latrunculin
E
Actin Speed(nm/s)
80
60
40
20
0
Outward
Inward
ns
ns
ns
ns
Primary CD4
Primary CD8
Jasplakinolide
Latrunculin

© The Author(s)

Figure EV3. Pharmacological interrogation of actin polymerization dynamics in generation of anterograde and retrograde flows.

(**A**) Snapshots of control or actin inhibitor treated T cells' synapses. The LifeAct distribution in mouse Primary CD4+ cells, and CD8 + T cells with and without treatment with 1 μM Jasplakinolide or 200 nM Latrunculin are shown in left panels. The middle panel shows the particle image velocimetry (PIV) images, and the right panel shows the temporal colour-coded images. Scale bar, 5 μm. (**B**) The ratio of the actin anterograde features are depicted in the graphs. The fraction of outward moving actin features are similar for CD4 and CD8 T cells. Treatment with Latrunculin A and Jasplakinolide does not significantly affect the ratio of outward moving actin features (*p*-values; Primary CD4+ vs. Primary control CD8+ cells: 0.566; Primary CD8+ control vs. Jasplakinolide treated cells: 0.392; Primary control CD8+ vs. Latrunculin A treated cells: 1.0; Jasplakinolide vs. Latrunculin treated CD8+ cells: 0.116) (No. of cells analyze $n \geq 5$ cells) (Data represented as Mean ± SEM). (**C**) The number of detected outward and inward moving features used for analysis in graph B were plotted separately (*p*-values of comparison between inward and outward features: CD4+ cells: 0.017; CD8+ cells: 0.312, Jasplakinolide treated CD8+ cells: 0.013; and Latrunculin treated CD8+ cells: 1.0). (Data represented as Mean ± SEM). (**D**) The speed of actin features in CD4+ and CD8 + T cells with and without treatment with Jasplakinolide or Latrunculin (*p*-values; Primary CD4+ vs. Primary control CD8+ cells: 0.207; Primary control CD8+ vs. Jasplakinolide treated cells: 0.035; Primary control CD8+ vs. Latrunculin-treated cells: 0.048, Primary CD8+ Jasplakinolide vs. Latrunculin treated cells: 0.033). (Data represented as Mean ± SEM) (**E**). The speed of outward and inward moving actin features from the data used in Graph D were plotted (*p*-values of comparison between inward and outward speeds are; CD4+ cells: 0.068; control CD8+ cells: 0.062; Jasplakinolide treated CD8+ cells: 0.275; and Latrunculin treated CD8+ cells: 0.156). All statistical analysis in (**B–E**) was using Mann–Whitney non-parametric two-tailed test. (Data represented as Mean ± SEM). Source data are available online for this figure.

© The Author(s)
